# Temporal synchronization during bilateral foot-tapping and its relationship with gait disturbance in patients with Parkinson’s disease

**DOI:** 10.3389/fneur.2026.1860090

**Published:** 2026-10-01

**Authors:** Atsuki Numata, Yasuo Terao, Naohiko Togashi, Kazuko Hasegawa, Kenichi Sugawara, Yoshikazu Ugawa, Toshiaki Furubayashi

**Affiliations:** 1 Physical Therapy Course, Department of Rehabilitation, Faculty of Medical Science and Welfare, Tohoku Bunka Gakuen University, Sendai, Japan; 2 Department of Medical Physiology, Faculty of Medicine, Kyorin University, Mitaka, Japan; 3 Department of Neurology, National Hospital Organization, Sagamihara National Hospital, Sagamihara, Japan; 4 Graduate Course of Health and Social Services, Kanagawa University of Human Services Graduate School, Yokosuka, Japan; 5 Department of System Neuroscience, Fukushima Medical University, Fukushima, Japan; 6 Department of Neurology, NHO Fukushima National Hospital, Sukagawa, Fukushima, Japan; 7 Graduate School of Health and Environmental Science, Tohoku Bunka Gakuen University, Sendai, Japan

**Keywords:** dysrhythmia, freezing of gait, gait rehabilitation, Parkinson’s disease, synchronized tapping, temporal process

## Abstract

**Objective:**

Previous studies using finger-tapping tasks have shown that patients with Parkinson’s disease (PD) exhibit a deficit of temporal processing linked to gait disturbance. In this study, we aimed to investigate abnormalities in lower-limb rhythmic control and their association with freezing of gait in PD using a bilateral foot-tapping task.

**Methods:**

Fifteen patients with PD and 25 healthy controls performed foot-tapping tasks synchronized with auditory tones at fixed intervals (500–2,400 ms). The tasks included unilateral and bilateral foot-tapping, with bilateral movements performed either in-phase (simultaneous) or antiphase (alternating). Data on synchronizing tapping error and inter-tap interval variability were analyzed and correlated with scores from clinical assessments, including the Unified Parkinson’s Disease Rating Scale and Freezing of Gait Questionnaire.

**Results:**

Patients with PD showed greater negative synchronizing tapping error (negative asynchrony) and higher inter-tap interval variability than did controls, especially in antiphase condition. Exploratory within-PD-group analyses suggested associations between clinical severity and abnormalities in bilateral foot-tapping performance.

**Conclusion:**

These findings indicate altered temporal accuracy and stability of rhythmic lower-limb movements in PD, particularly during bilateral antiphase foot-tapping. Bilateral foot-tapping measures may help characterize lower-limb timing deficits that are relevant to gait rehabilitation in PD.

## 1 Introduction

The basal ganglia (BG) play a crucial role in controlling motor functions and gait and are significantly involved in temporal processing. The BG is particularly important for the cognitive processing of timing, especially in perceiving time intervals longer than a second (supra-second timing) and timing based on rhythmic beats (beat-based timing) (1–7).

Deficits in central temporal processing are commonly observed in Parkinson’s disease (PD), alongside its characteristic motor symptoms (8–10). PD is characterized by the progressive degeneration of dopaminergic neurons in the nigrostriatal pathway, leading to dysfunction of the BG circuitry that underlies motor and temporal processing impairments (11). These temporal deficits have been robustly demonstrated in studies using finger-tapping tasks (8, 9, 12–14). Functional abnormalities within the BG-thalamocortical network, which consists of brain regions involved in motor preparation and programming, have been associated with timing impairments (15, 16). Furthermore, the link between motor symptoms and disrupted temporal processing supports a potential connection between freezing of gait (FOG)—a phenomenon characterized by a transient inability to initiate or maintain effective forward stepping despite the intention to walk—and these deficits in patients with PD (17, 18).

Patients with PD often exhibit impaired interlimb coordination (19). Research on bilateral coordination impairments has primarily focused on upper limb movements, especially during rhythmic, repetitive tasks. In this context, antiphase movements, in which the limbs move in opposite directions, are more difficult to perform than in-phase movements, in which the limbs move in the same direction (20–22). The supplementary motor area, which plays a key role in antiphase movements relative to in-phase movements (23), receives inputs from the BG via the thalamus, as part of the motor loop. Functional abnormalities in the supplementary motor area have been well documented in PD neuroimaging studies (24–26). Antiphase movements in PD are characterized by reduced activity in the BG and supplementary motor area, accompanied by increased motor cortical and cerebellar activation (23).

During rhythmic, cyclic movements of the upper limbs, a phenomenon called phase transition has been observed, in which, as the frequency of movement increases, bilateral antiphase movements shift to in-phase movements (27). In contrast, this shift does not occur during lower-limb movements, in which rhythmic stability in antiphase motion is preserved even at high frequencies of movement (28). To investigate this discrepancy, Numata et al. used a synchronized tapping task under three movement phase conditions—unilateral, in-phase, and antiphase—to compare the accuracy and temporal stability of tapping rhythms during bilateral finger and ankle joint movements in healthy young adults (29). They found that rhythm stability was significantly greater during bilateral antiphase foot movements than during unilateral and in-phase foot movements. In contrast, no significant differences were observed among the phase conditions during upper limb movements. These results suggest that bilateral antiphase movements of lower limbs may rely on control mechanisms that differ from those of the upper limbs, particularly in achieving rhythmic stability. However, the neural mechanisms underlying this characteristic remain uncertain, and the extent to which brainstem–spinal rhythm-generating mechanisms contribute to voluntary foot-tapping performance is unknown.

Abnormal network activity in the BG, cerebellum, and primary motor cortex during lower-limb movement has been observed in patients with PD compared with healthy individuals (30). Unilateral and bilateral finger-tapping tasks have been widely used in PD research to assess temporal processing deficits associated with the dysrhythmic characteristic of PD (9, 14, 17–19, 31–33). However, the connection between central temporal processing deficits and FOG in PD has not yet been explored using a bilateral foot-tapping task.

Given the distinct neural control mechanisms governing rhythmic motor functions in the upper and lower limbs, a bilateral foot-tapping task was employed, as it is more pertinent to gait dysfunction and related temporal processing deficits in PD. In this study, gait disturbances in PD were postulated to arise from the combined effects of motor impairments and temporal processing deficits. We aimed to evaluate lower-limb rhythm generation in patients with PD and age-matched healthy adults through a bilateral foot-tapping task, by analyzing the relationship between FOG severity and rhythmic function. We hypothesized that patients with PD would show reduced accuracy and stability of rhythmic lower-limb movements, particularly during bilateral antiphase foot-tapping, and that these abnormalities would be associated with disease severity and FOG symptoms. Additionally, we aimed to explore the relationship between motor and gait disturbances in PD and dysrhythmia, by correlating the foot-tapping task performance with the scores of the Unified Parkinson’s Disease Rating Scale (UPDRS) and Freezing of Gait Questionnaire (FOG-Q).

## 2 Materials and methods

### 2.1 Participants

Fifteen patients with PD with Hoehn–Yahr stage ≤ III, who were able to walk independently, and 25 age-matched healthy controls (HCs) participated in this study (Table 1). The HCs had no history of neurological or orthopedic diseases. Foot preference was evaluated using Chapman’s questionnaire (34), and all participants were found to be right-foot dominant. The Mini-Mental State Examination (MMSE) was used to assess cognitive function. For patients with PD, parts II and III of the UPDRS were used to assess motor function, and the FOG-Q was used to assess the severity of FOG. These clinical assessments were conducted during the same study period as the synchronized foot-tapping task. All patients with PD were assessed in the ON-medication state while they received their usual antiparkinsonian medications. Testing was generally conducted approximately 2–3 h after the patients had taken their usual antiparkinsonian medication following a meal. However, the exact interval between the most recent medication dose and experimental assessment was not strictly standardized across participants. Written informed consent was obtained from all participants, and the study was approved by the Ethics Committee of Tohoku Bunka Gakuen University (No. 20–03).

**Table 1:** Characteristics of participants.

Characteristics	PD (mean ± SD)	HC (mean ± SD)	*p*-value
Sex (n)	Male: 10, female: 5	Male: 14, female: 11	-
Age (y)	72.1 ± 8.9	72.1 ± 5.7	0.982
MMSE score	27.7 ± 1.6	28.8 ± 1.7	0.090
Hoehn–Yahr stage (*n*)	I: 1, II: 6, III: 8	-	-
UPDRS score (*n* = 14)	Part II: 5.6 ± 4.2, Part III: 12.0 ± 7.2	-	-
FOG-Q score (*n* = 14)	10.5 ± 5.1	-	-
LEDD (mg)	583.4 ± 242.7	-	-
Medication state	ON (*n* = 15)		

PD, Parkinson’s disease; SD, standard deviation; HC, healthy control; MMSE, Mini-Mental State Examination; UPDRS, Unified Parkinson’s Disease Rating Scale; FOG-Q, Freezing of Gait Questionnaire; LEDD, Levodopa equivalent daily dose.

### 2.2 Experimental procedure

The participants performed a synchronized tapping task in which they had to press a button with their feet (foot-tapping task) in synchrony with sounds presented at regular intervals [interstimulus intervals (ISIs)]. The tasks were conducted in three movement phases: (i) unilateral movement of the dominant foot (Uni), (ii) bilateral synchronous movement in phase, alternating between bilateral dorsiflexion and plantarflexion in the same direction (bilateral in-phase movement: Bi-in), and (iii) bilateral synchronous movement of the ankles in antiphase, alternating unilateral dorsiflexion and plantarflexion of each ankle but in the opposite direction (bilateral antiphase movement: Bi-anti) (29). The participants sat comfortably on a chair and placed the ball of their foot on a button. In the bilateral condition, each ball of their foot pressed a different button. Figure 1A shows a schematic diagram of these conditions.

**Figure 1:**
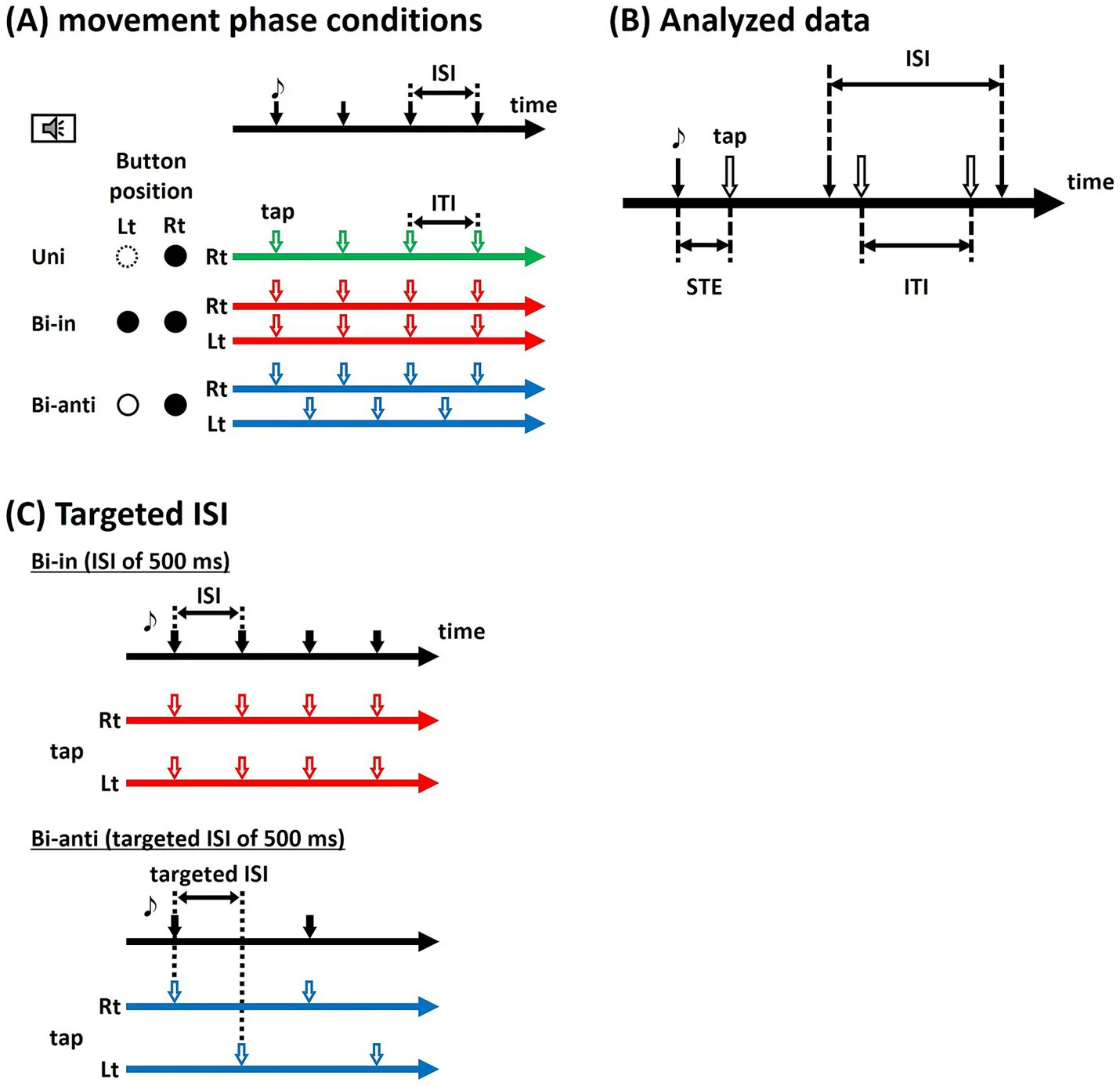
Tapping task**. (A)** Participants were instructed to tap the button in synchrony with sounds presented at fixed interstimulus intervals (ISIs) using either one foot (Uni condition) or both feet (Bi-in condition, moving both ankles simultaneously; and Bi-anti condition, moving both ankles alternately). **(B)** The timing of the tap and presented sound [synchronizing tapping error (STE)] and the time interval between successive taps [inter-tap interval (ITI)] were evaluated. **(C)** In the Bi-anti condition, the targeted ISI was defined to match the timings of the taps on both sides with the other conditions.

In the Bi-anti condition, the participants were required to tap their dominant foot in synchrony with each auditory tone (on-beat foot-tapping) and tap the non-dominant foot midway between successive tones, without a separate auditory cue (off-beat foot-tapping). Thus, the tapping of the two feet alternated with an approximately 180° phase relationship while a single auditory stream served as the external temporal reference. We deliberately avoided providing separate pacing tones for each limb because dual-tone pacing could increase task complexity, particularly at faster tapping rates. This decision was made based on a previous study that reported poorer tapping performance under such externally paced bilateral coordination conditions (21). Although this procedure made the task partly similar to a continuation task for the non-dominant limb, previous studies have shown differences between synchronization and continuation performance while reporting broadly comparable patterns of impairment in PD (8, 35–37).

In previous studies comparing bilateral in-phase and antiphase movements of the fingers and feet, auditory stimuli corresponding to movements on both sides were often presented and the cyclicity of finger flexion–extension or ankle plantarflexion–dorsiflexion were evaluated (27, 28, 38, 39). In such paradigms, the timing for tapping each limb can be specified by external cues, and an uncued off-beat tap is not required. In contrast, this study was designed to evaluate the timing of discrete foot-tapping during bilateral lower-limb rhythm generation. Accordingly, in the Bi-anti condition, the tapping of the dominant foot was directly synchronized with the auditory tones, whereas the non-dominant foot generated an intermediate off-beat tap between successive tones. Consequently, each auditory ISI contained two alternating bilateral tap timings in the Bi-anti condition, whereas the Uni and Bi-in conditions contained one externally defined tap timing per ISI. This task configuration was adopted in accordance with our previous foot-tapping studies (29, 40). Therefore, the Bi-anti condition differed qualitatively from conventional bilateral cyclic-movement paradigms; this difference should be considered when interpreting the results.

The tasks were performed in 18 blocks, representing all combinations of the 6 different ISIs used (500, 600, 900, 1,200, 1,800, and 2,400 ms) and the 3 movement phases (Uni, Bi-in, and Bi-anti). During each block, 110 acoustic sounds were presented. The ISI and movement phase for each block were randomly selected from the six ISIs and three movement phases, using a random number table, in a counterbalanced manner. Participants were instructed to synchronize their taps with the tempo of the sounds. They typically established an approximate tapping pace within the first 10 taps, guided by the auditory cues. To avoid muscle fatigue, the participants took a brief break (approximately 1–2 min) between blocks. The acoustic sounds (click sounds) were generated by converting electrical signals from an electrical stimulator (STEN-3401, Nihon Kohden, Japan) into acoustic signals using an audio monitor (MODEL 3300, A-M Systems, Inc., Sequim, WA, USA). The sound was set at a volume at which it could be readily heard (a single-pulse sound with a duration of 1 ms, 50-dB sound pressure level) but would not startle the participants. The acoustic sound stimulation and tapping times were recorded using a laboratory computer through an A/D converter (PowerLab 8/35, AD Instruments Pty Ltd., Sydney, Australia) for offline analyses (LabChart, AD Instruments Pty Ltd.). Additionally, we evaluated whether the precision of the timings recorded by the computer program matched those obtained by visual confirmation of the recorded sound waves.

Following completion of the synchronized foot-tapping task, in a separate session, the participants performed a simple reaction time (SRT) task using their dominant foot to evaluate their simple sensorimotor function. This task confirmed whether their reaction times fell within the normal, healthy range (approximately 200 ms). In addition, the SRT was measured to determine whether delayed tapping at longer ISIs (2,400–4,800 ms) corresponded to each participant’s own SRT distribution (41, 42). In other words, the task was performed to determine whether tapping was performed based on temporal prediction of the upcoming sound or in reaction to the presented sound. The participants were instructed to press the button using the ball of their foot as fast as possible after an acoustic “go” signal was presented. The ISI of the signals varied between 4,500 and 5,500 ms across trials, as in our previous studies (9, 29). Thirty trials were performed within each block.

### 2.3 Data analysis

Data from the dominant foot were used for all analyses. As the on-beat tap (Uni, Bi-in) and off-beat tap (Bi-anti) coexisted for tapping using the non-dominant foot, only the data on the timing of the dominant-side taps were analyzed (29, 40). After discarding the data of the first 10 taps, we analyzed the data of 100 taps recorded during each block, including the time of the presented sound and timing of the tap. For data analysis, we calculated two onset-to-onset measures in each trial (Figure 1B): the onset time difference between the presented sound and taps (synchronizing tapping error [STE]) and the inter-tap interval (ITI), or the interval between consecutive taps. If the tapping preceded the presented sound, the STE took a negative value (negative asynchrony), a phenomenon in which the tap slightly precedes the sound stimulus (43–45). Conversely, when the tap followed the sound, the STE took a positive value. Irregular data (e.g., participants who missed taps to the sounds or had two consecutive taps) were excluded as outliers. We also excluded taps that could not be associated with either the preceding or following sounds. Trials in which the participants failed to synchronize their taps to the sounds owing to excessive time shifts caused by delayed or premature tapping were excluded from the analysis. For statistical analyses, we calculated the mean STE and coefficient of variation (CV) of the ITI. The CV was calculated by dividing the standard deviation of the ITI by the mean value of the ITI for each block.

In this study, the timing of tapping of the dominant foot was used as the reference. In the antiphase condition, tapping of the non-dominant foot occurred off-beat, effectively doubling the number of tap time points (i.e., including dominant and non-dominant taps) compared with other phase conditions. A more accurate timing of tapping may be achieved by subdividing the interval into two by tapping at an intermediate time point during the ISI (46). To control for this difference in the number of taps in the Bi-anti condition and the Uni and Bi-in conditions, we performed a different analysis in which we matched the number of tap timings on both sides between the Bi-anti condition and the Uni and Bi-in conditions (Figure 1C). In this analysis, we defined the “targeted ISI” as the time interval between the pacing tones for the dominant and non-dominant limbs, rather than the interval between the tones as before (29). In the Bi-anti condition, participants were required to tap in synchrony with the tones presented at the targeted ISI, alternating between their dominant and non-dominant feet. Meanwhile, in the Uni and Bi-in conditions, tapping was performed using the unilateral foot/bilateral feet, in synchrony with the pacing tones presented at the same ISI. Thus, the number of tap timings for both sides was matched. For example, in the Bi-anti condition, an ISI of 900 ms in the Uni and Bi-in conditions corresponded to an ISI of 1,800 ms, with the targeted ISI defined as 900 ms. Similarly, a 1,200 ms ISI in the Uni and Bi-in conditions corresponded to an ISI of 2,400 ms in the Bi-anti condition, with the targeted ISI defined as 1,200 ms. Thus, two targeted ISIs—900 and 1,200 ms—were selected based on the ITI.

To determine whether the hastening phenomenon emerged during synchronized tapping in patients with PD, particularly at the beginning of synchrony, we calculated the regression coefficients between changes in the STE and tap number for the first 20 taps from the beginning of the analysis. The STE was smoothed using a 3-point moving average. The Bi-anti condition at ISIs of 1,800 and 2,400 ms exhibited a progressive increase in negative STE (i.e., tapping was performed progressively earlier), compared with the other movement phase conditions (Figures 2B,C). This trend was particularly pronounced during the first 20 taps, after which the STE fluctuated while showing a gradual overall decrease. Notably, a previous study (9) also reported a rapid increase in negative asynchrony during the initial phase of synchronization. Therefore, we considered the changes in the STE during the first 20 taps as reflecting a characteristic feature of rhythm generation in PD and therefore restricted the subsequent analysis to this interval.

**Figure 2:**
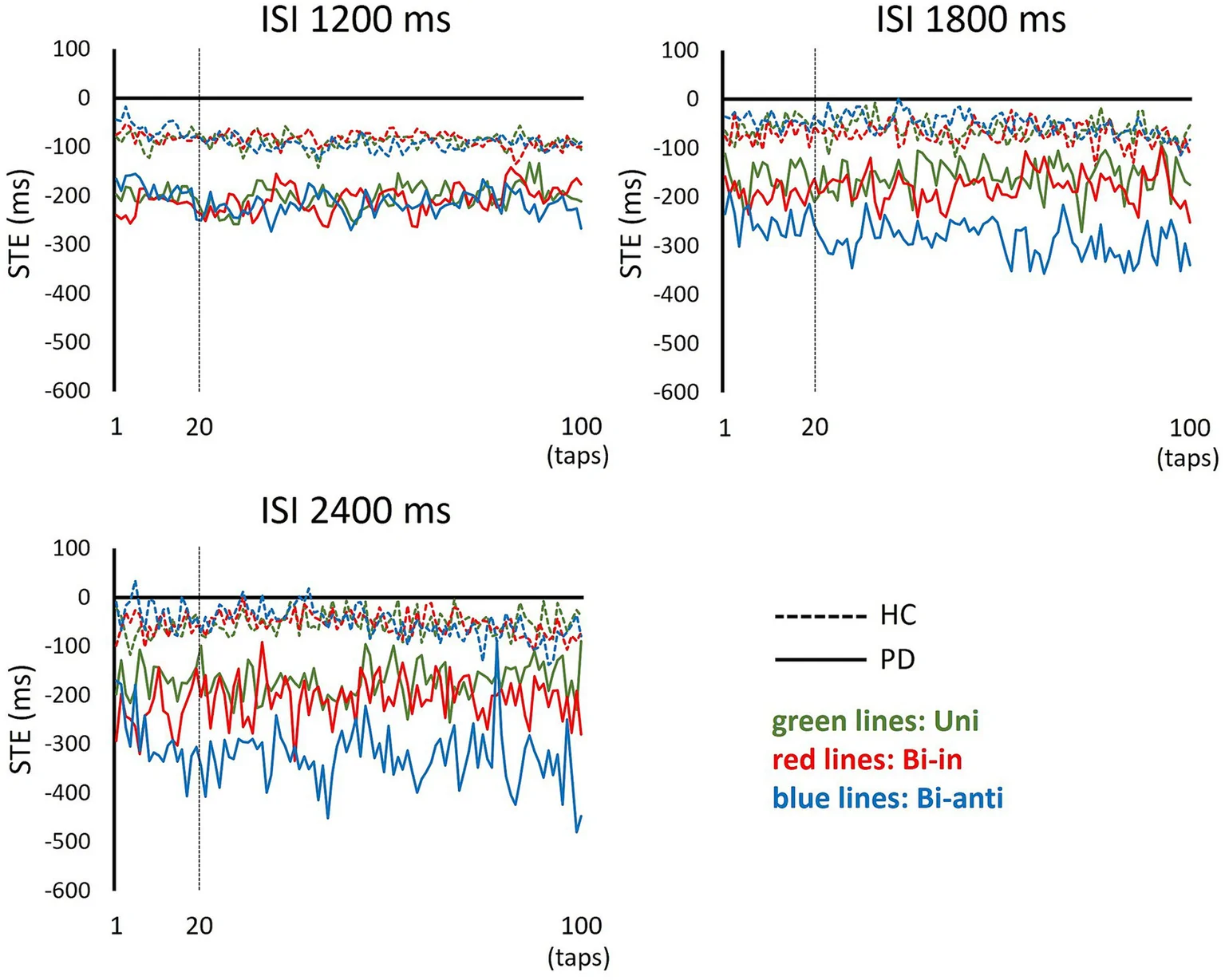
Time course of the synchronizing tapping error (STE) of healthy controls (HCs) and patients with Parkinson’s disease (PD). The STE of each tapping (vertical axis) is plotted against the tap number (horizontal axis). Solid lines indicate HCs, whereas dashed lines indicate patients with PD. The colors of the lines indicate the Uni (green), Bi-in (red), and Bi-anti (blue) conditions. Regression coefficients used for the hastening analysis were calculated from the data on the initial 20 taps.

For the SRT task, the reaction time was measured from the time of sound presentation to the time of tapping. The mean and standard deviation of the SRT were calculated. Furthermore, to examine whether reactive tapping increases with increasing ISI, taps with latencies exceeding the 95th percentile of each participant’s SRT distribution were defined as “delayed taps,” based on the study by Tokushige et al. (9). The number of delayed taps for each participant was counted, and the ratio of delayed taps was calculated as the proportion of delayed taps to the total number of taps.

### 2.4 Statistical analyses

Age and MMSE scores were compared between groups using an unpaired t-test. The SRT was compared between the PD and HC groups using an unpaired t-test. For ISIs of 500, 600, and 900 ms, any block in which at least one participant was unable to synchronize the taps with the auditory stimuli was excluded in its entirety from all analyses (Supplementary Table 1). Therefore, data from the blocks with ISIs of 1,200, 1,800, and 2,400 ms were included in the statistical analysis of the mean STE and those from the blocks with ISIs of 900 (Uni and Bi-in), 1,200 (Uni, Bi-in, and Bi-anti), 1,800 (Uni, Bi-in, and Bi-anti), and 2,400 (Uni, Bi-in, and Bi-anti) ms were used for the analysis of the CV of the ITI (Supplementary Tables 2, 3). Data from 15 patients with PD and 23 HC participants were included in the targeted ISI analysis.

Data from the foot-tapping task (mean STE, CV of ITI, and proportion of delayed taps) were analyzed using a three-way analysis of variance (ANOVA), with the group (PD and HC) as the between-participants factor and the movement phase condition (Uni, Bi-in, and Bi-anti) and ISI (1,200, 1,800, and 2,400) as within-participant factors. Mauchly’s test of sphericity was used to assess the assumption of sphericity using repeated-measures ANOVA. Where necessary, the Greenhouse–Geisser correction was used to correct violations of the assumption of sphericity. Post-hoc analyses were performed using two-tailed t-tests with Bonferroni corrections to detect significant differences between various comparisons. A separate three-way ANOVA was performed on the CV of the ITI using the group as the between-participants factor and the movement phase and targeted ISI (900 and 1,200 ms) as within-participant factors, based on matched tap timings in the Bi-anti condition (targeted ISI) (29, 40).

To examine the hastening phenomenon, a third three-way ANOVA was conducted using the regression coefficient for the initial 20 taps as the dependent variable, with the group as the between-participants factor and the movement phase and ISI (1,200, 1,800, and 2,400 ms) as within-participant factors.

Spearman’s rank correlation coefficients were used to examine the associations between foot-tapping performance (mean STE, CV of the ITI, regression coefficient, SRT, and proportion of delayed taps) and clinical measures (Hoehn–Yahr stage, UPDRS parts II and III scores, and FOG-Q score) in patients with PD. For one patient, the data (UPDRS and FOG-Q) required for the correlation analyses were missing; therefore, these analyses were performed using complete data from 14 patients. As the correlation analyses were exploratory and hypothesis-generating, the *p* values were not adjusted for multiple comparisons and were interpreted cautiously.

A sensitivity analysis based on the bivariate correlation model was conducted using G*Power version 3.1 to estimate the minimum detectable correlation given the available sample size. As G*Power does not provide a dedicated sensitivity analysis for Spearman’s rank correlation, the bivariate normal correlation model was used as an approximation. With a total sample size of 14, a 2-sided α level of 0.05, and a statistical power of 0.80, the minimum detectable correlation was approximately |r| = 0.66. All statistical analyses were performed using commercial software (IBM SPSS 27 for Windows, SPSS, Chicago, IL, USA). Statistical significance was set at *p* < 0.05. To reduce the probability of type-I errors, the significance level for three-way ANOVA was adjusted using the standard Bonferroni correction (α divided by the total number of tests): 0.05/7 = 0.007 (47). Therefore, for the three-way ANOVA, *p* < 0.007 was considered statistically significant.

## 3 Results

### 3.1 Characteristics of participants

The participants’ characteristics are listed in Table 1. No significant differences were observed between the groups in terms of age or the MMSE scores. The mean FOG-Q score was 10.5 ± 5.1, indicating the presence of FOG in the PD group.

### 3.2 Reaction time in the SRT task

An unpaired t-test revealed no significant differences in the SRT between the PD and HC groups (*p* = 0.065) (see section 2.2).

### 3.3 Synchronized foot-tapping task

The ANOVA results (Supplementary Tables 4, 5) focused on the main effects and interactions of the group factors.

Regarding the mean STE (Supplementary Table 2 and Figures 3A, 4A), three-way ANOVA revealed a significant main effect of group. Additionally, a significant interaction was observed between the group and movement phase. Post-hoc analysis revealed that the mean STE was significantly more negative in the PD group (−217.0 ± 16.1 ms) than in the HC group (−66.4 ± 6.7 ms, *p* < 0.001). Post-hoc analyses performed within the groups revealed that the mean STE was significantly smaller in the Bi-anti condition than in the other conditions in the PD group (Uni: −177.1 ± 152.3 ms, Bi-in: −203.0 ± 171.1 ms, Bi-anti: −271.0 ± 222.2 ms; vs. Uni: *p* < 0.001, vs. Bi-in: *p* = 0.021). In contrast, no significant differences were observed among the movement phase conditions in the HC group (Uni: −67.2 ± 100.9, Bi-in: −69.8 ± 95.9, Bi-anti: −62.28 ± 104.8). Post-hoc analyses performed within the groups across different movement phase conditions indicated that the mean STE in each condition was significantly smaller in the PD group than in the HC group (Uni: *p* = 0.003, Bi-in: *p* = 0.001, Bi-anti: *p* < 0.001). Regarding the CV of the ITI (Supplementary Table 2 and Figure 3B), a three-way repeated-measures ANOVA revealed no significant main effect of the group nor any significant interactions involving the group factor.

**Figure 3:**
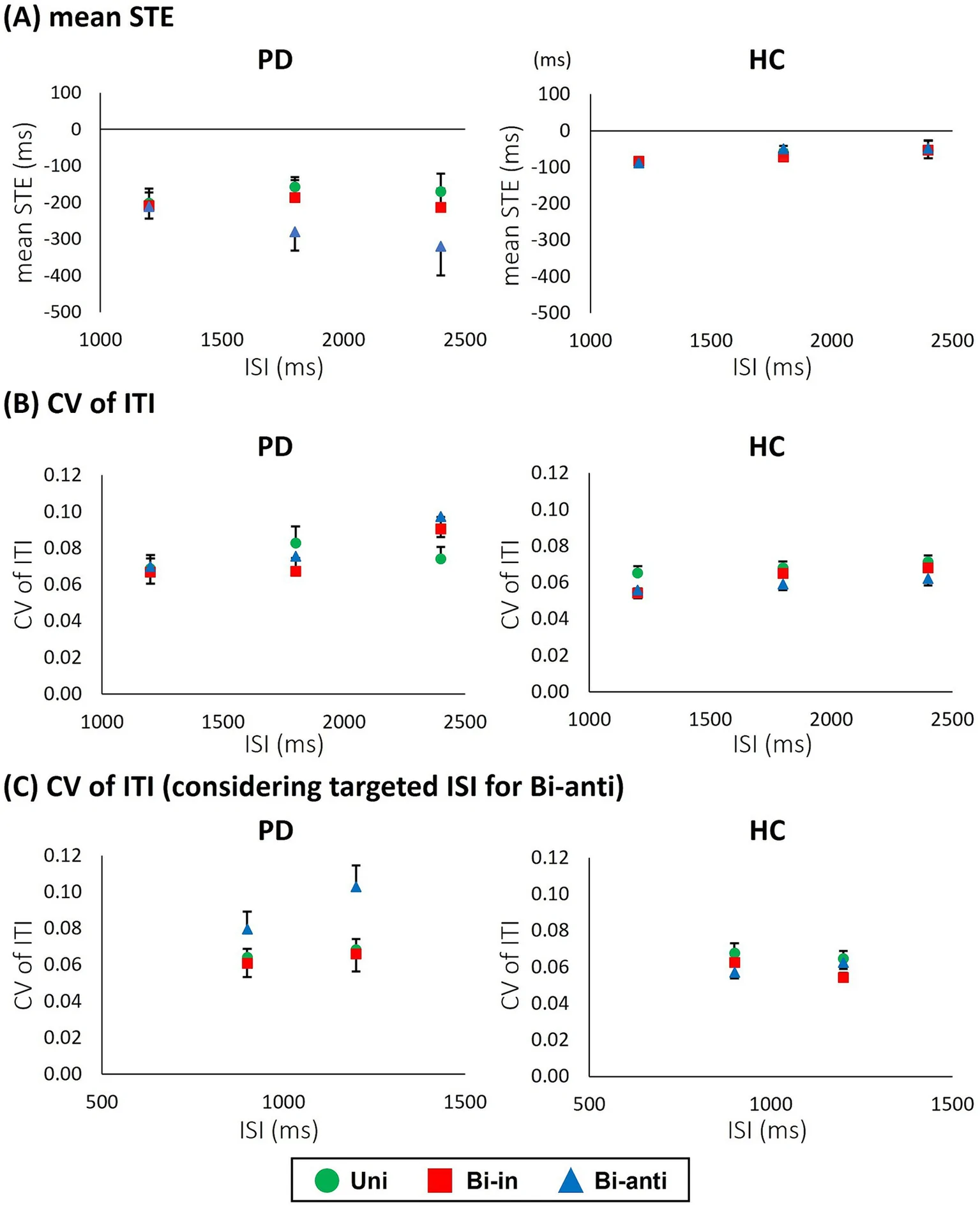
Differences in various parameters for each movement phase condition in the foot-tapping task. **(A)** The mean synchronizing tapping error (STE) in the Uni (circles), Bi-in (rectangles), and Bi-anti (triangles) conditions for each interstimulus interval (ISI) in each group. The error bars indicate standard error. **(B)** The coefficient of variation (CV) of the inter-tap interval (ITI) for each ISI. **(C)** The CV of the targeted ISI for each ISI.

**Figure 4:**
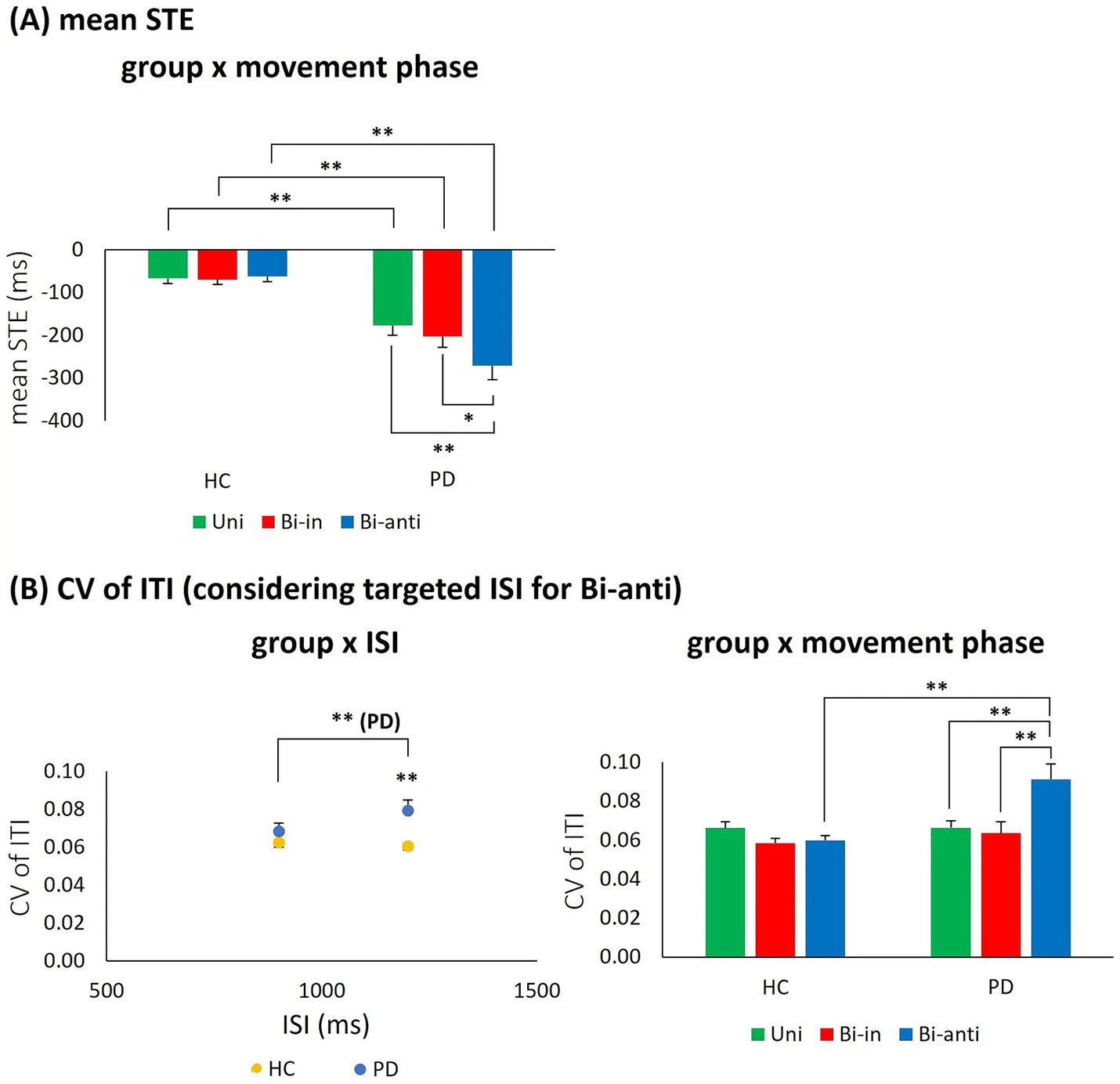
Difference in the mean synchronizing tapping error (STE) and coefficient of variation (CV) of the inter-tap interval (ITI) (considering the targeted interstimulus interval [ISI] for the Bi-anti condition) between groups (results of multiple comparisons). **(A)** The mean STE of the Uni, Bi-in, and Bi-anti conditions for each group. The error bars indicate standard error. **(B)** The CV of the ITI (considering the targeted ISI for the Bi-anti condition) for each ISI (left) and movement phase condition (right). Asterisks indicate significant differences (**p < 0.01).

### 3.4 Changes in the CV of the ITI considering the timing of the off-beat tap in the bi-anti condition (targeted ISI)

A three-way repeated-measures ANOVA (Supplementary Table 3, Figures 3C, 4B) revealed no significant main effect of the group. However, significant interactions were observed between the group and movement phase and between the group and ISI.

Post-hoc analyses performed within the groups showed that the CV of the ITI was significantly larger in the Bi-anti condition than in the other conditions in the PD group (Uni: 0.066 ± 0.020, Bi-in: 0.063 ± 0.033, Bi-anti: 0.091 ± 0.042; vs. Uni: *p* = 0.003, vs. Bi-in: *p* = 0.003). In contrast, no significant differences were observed among the movement phase conditions in the HC group (Uni: 0.066 ± 0.022, Bi-in: 0.058 ± 0.017, Bi-anti: 0.060 ± 0.016). Post-hoc analyses under different movement phase conditions indicated that the CV of the ITI in the Bi-anti condition was significantly larger in the PD group than in the HC group (*p* = 0.001).

Post-hoc analyses performed within the groups showed that, in the PD group, the CV of the ITI was significantly higher at an ISI of 1,200 ms than at 900 ms (900 ms: 0.068 ± 0.030, 1,200 ms: 0.079 ± 0.039, *p* = 0.003). In contrast, no significant difference between ISIs was observed in the HC group (900 ms: 0.062 ± 0.020, 1,200 ms: 0.060 ± 0.017). Post-hoc analyses across different ISIs indicated that the CV of the ITI at 1,200 ms was significantly higher in the PD group than in the HC group (*p* = 0.006).

### 3.5 Changes in the STE during the foot-tapping task

Supplementary Table 6 presents the proportion of delayed taps at each ISI and under each movement condition, separately for each group. In the HC group, the proportion of delayed taps was greater than 5% at an ISI of 1,800 ms. In the PD group, it surpassed 5% at an ISI of 2,400 ms but remained lower than that in the HC group. Three-way repeated-measures ANOVA showed no significant main effect of the group nor any significant interactions involving the group factor (Supplementary Table 4). Post-hoc analysis demonstrated that the proportion of delayed taps was significantly higher at an ISI of 2,400 ms than at other ISIs (ISI of 1,200 ms: 0.6 ± 2.1%; 1,800 ms: 6.7 ± 16.1%; 2,400 ms: 13.8 ± 24.6%; vs. 1,200 ms: *p* = 0.003; vs. 1,800 ms: *p* = 0.001).

Figure 2 shows the time course of the mean STE across 100 successive taps at each ISI and in each movement phase condition in the PD and HC groups. The regression coefficients calculated from the data on the initial 20 taps for both groups across all movement phase conditions and ISIs are presented in Supplementary Table 7. In the Bi-anti condition, the regression coefficients were −3.10 ± 14.68, −1.18 ± 6.11, and −7.77 ± 15.37 in the PD group and −2.19 ± 7.29, −0.84 ± 3.44, and −1.82 ± 9.98 in the HC group at ISIs of 1,200, 1,800, and 2,400 ms, respectively. A three-way repeated-measures ANOVA of the regression coefficients showed no significant main effect of group or interactions involving the group factor (Supplementary Table 5). Thus, the regression coefficients did not differ significantly between the PD and HC groups.

Exploratory Spearman’s rank correlation analyses were performed using the data from the 14 patients with PD with complete data (Supplementary Table 8 and Figure 5). In the Uni and Bi-in conditions, no nominally significant associations were observed between the regression coefficient and motor or gait impairment. In the Bi-anti condition, nominally significant negative correlations were observed between the regression coefficient and the Hoehn–Yahr stage, UPDRS parts II and III scores, and FOG-Q score at an ISI of 1,800 ms, whereas nominally significant positive correlations were observed with the UPDRS parts II and III scores at an ISI of 2,400 ms. These *p* values were not adjusted for multiple comparisons.

**Figure 5:**
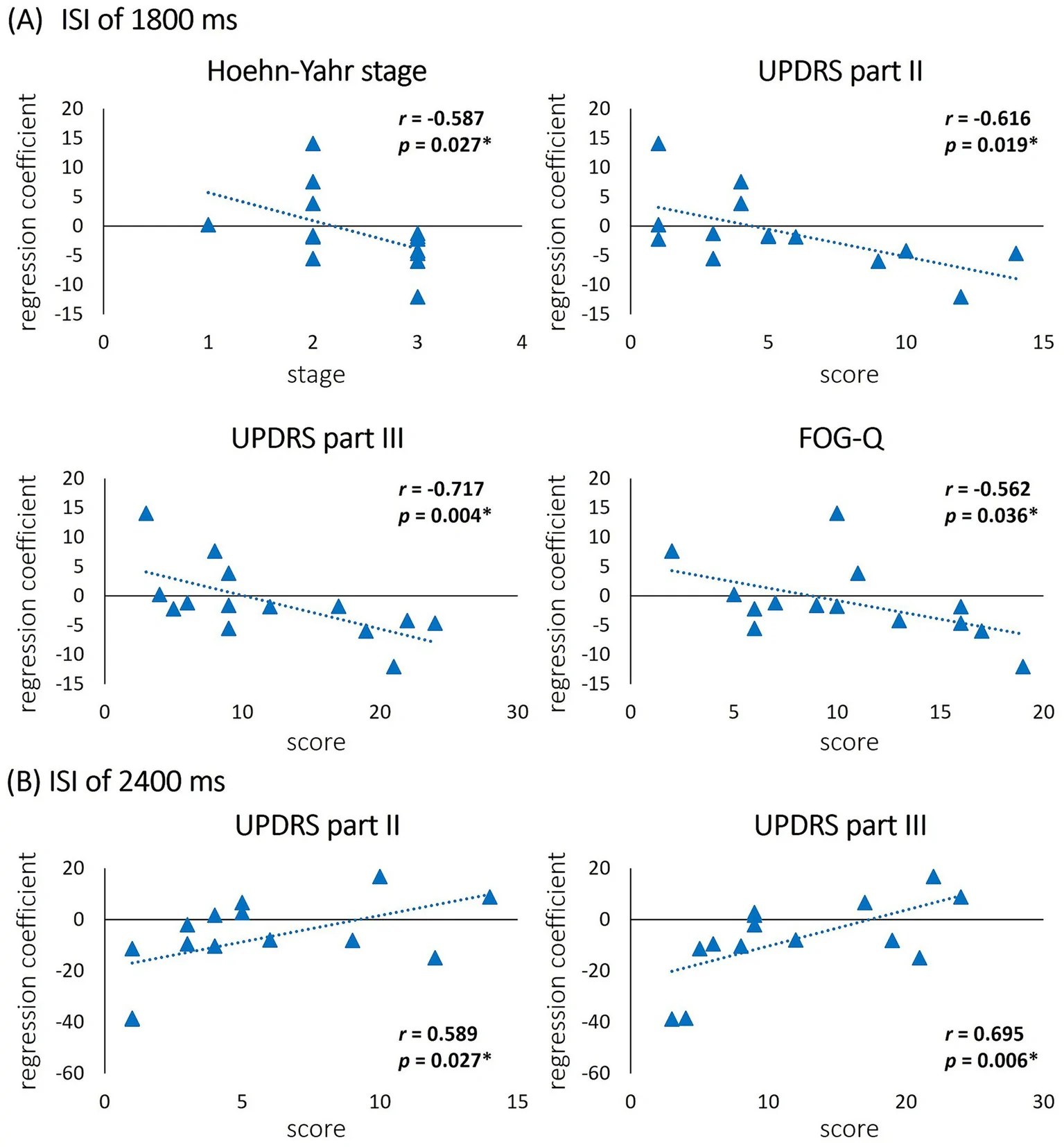
Relationship between regression coefficient and motor and gait impairments in patients with Parkinson’s disease (PD). The scattergram of the regression coefficient at interstimulus intervals (ISIs) of 1,800 ms **(A)** and 2,400 ms **(B)** in the Bi-anti condition is plotted against the Hoehn–Yahr stage, and the Unified Parkinson’s Disease Rating Scale (UPDRS) part II, UPDRS part III, and Freezing of Gait Questionnaire (FOG-Q) scores. Each data point represents an individual patient. Correlation analyses included data from 14 patients with complete data. Asterisks indicate nominally significant correlations (*uncorrected *p* < 0.05); the *p* values are not adjusted for multiple comparisons.

### 3.6 Relationship between foot-tapping performance and motor and gait impairments in PD

Supplementary Table 9 and Figure 6 show the results of the exploratory Spearman’s rank correlation analyses between foot-tapping performance and motor and gait impairments in the 14 patients with PD with complete data. Nominally significant associations were observed between the Hoehn–Yahr stage and CV of the ITI at an ISI of 1,800 ms in the Bi-in condition; between the UPDRS parts II and III scores and mean STE at an ISI of 1,200 ms; and between the FOG-Q score and mean STE at ISIs of 1,200 and 1,800 ms as well as the CV of the ITI at ISIs of 1,800 and 2,400 ms in the Bi-in condition. No other nominally significant associations were observed. The reported *p* values were not adjusted for multiple comparisons. A sensitivity analysis was also performed after excluding the data from a single patient with Hoehn–Yahr stage I (Supplementary Table 10). The direction of all reported associations was preserved, and the correlation coefficients generally remained moderate to large; however, several associations no longer reached the nominal *p* < 0.05 threshold after the exclusion.

**Figure 6:**
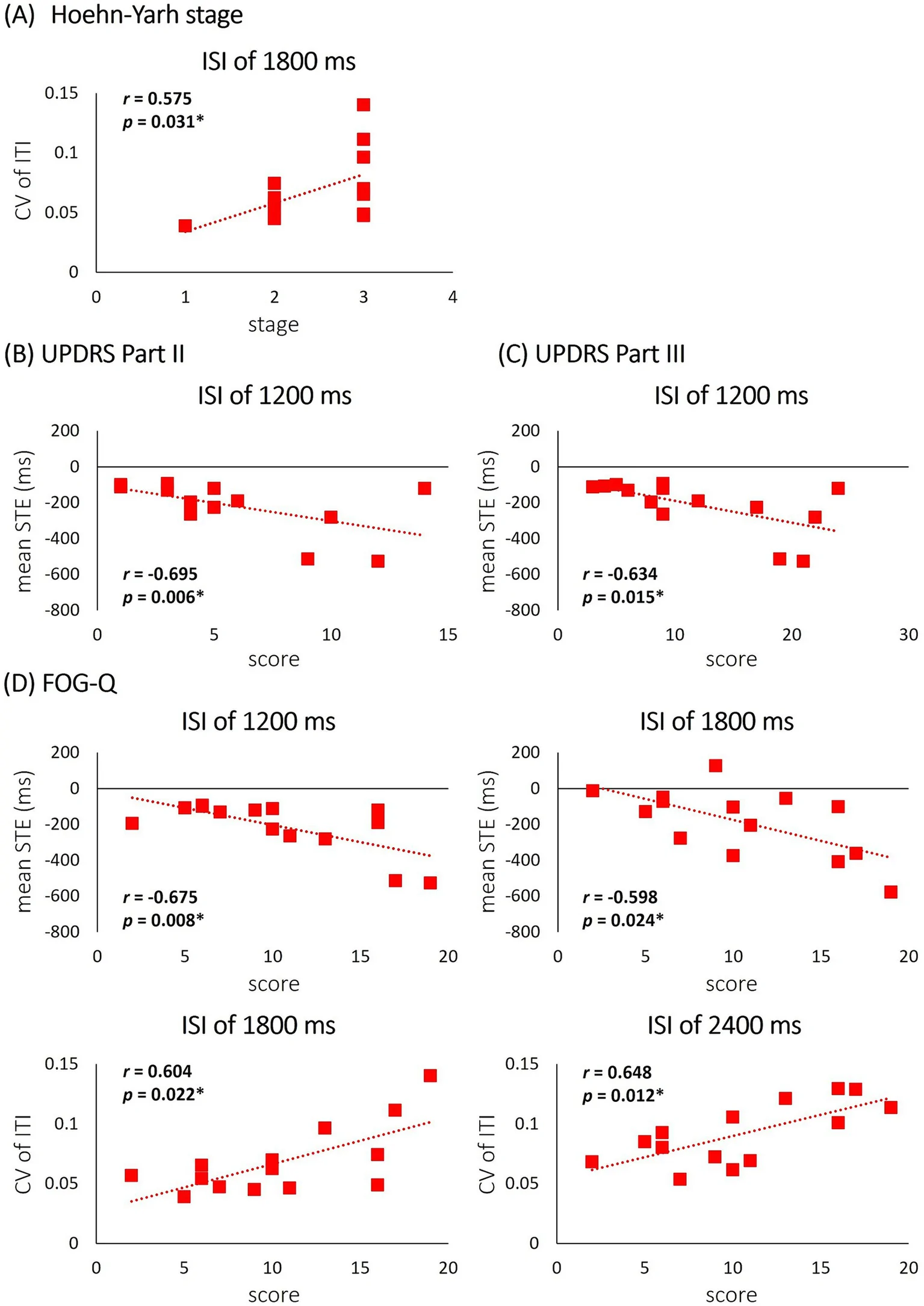
Relationship between tapping performance and motor and gait impairments in patients with Parkinson’s disease (PD). Scattergram of tapping performance [mean synchronizing tapping error (STE), coefficient of variation (CV) of the inter-tap interval (ITI)] in the Bi-in condition is plotted against the Hoehn–Yahr stage **(A)**, and the Unified Parkinson’s Disease Rating Scale (UPDRS) part II **(B)**, UPDRS part III **(C),** and Freezing of Gait Questionnaire (FOG-Q) **(D)** scores. Each data point represents an individual patient. Correlation analyses included data from 14 patients with complete data. Asterisks indicate nominally significant correlations (*uncorrected *p* < 0.05); the *p* values are not adjusted for multiple comparisons.

## 4 Discussion

In this study, we characterized the abnormalities of lower-limb rhythm generation in PD and explored their relationship with FOG and the underlying mechanisms. Patients with PD showed greater negative STE (negative asynchrony) and higher ITI variability than HCs did, especially in the Bi-anti condition. Tapping deficits in the Bi-in condition and hastening in the Bi-anti condition correlated with disease progression.

The mean STE in the PD group was significantly more negative than that in the HC group, indicating negative asynchrony with taps preceding auditory stimuli, which was particularly marked in the Bi-anti condition. In contrast, the mean STE of the HC group fell within the normal range of negative asynchrony reported in previous studies (43–45, 48). These results indicate temporal underestimation and/or hastening in the PD group.

Dopamine deficiency in PD causes distorted time perception (10, 49). This distortion may improve with dopaminergic therapy or deep-brain stimulation (8, 50), although some studies have reported no effect of these treatments (51, 52). The pronounced negative asynchrony in the Bi-anti condition in PD may be linked to time underestimation. The hastening phenomenon (progressively earlier tapping) likely results from shortened ITIs. Tokushige et al. (9) reported greater hastening during synchronized unilateral finger-tapping in an early PD group (UPDRS part III score ≤ 15) than in an advanced PD group (UPDRS part III score >15) and HCs, whereas the advanced PD group showed delayed taps, likely because of reactive responses. In our study, delayed taps increased with longer ISIs but showed no significant group or movement phase differences. The mean STE results indicated a significant tendency for earlier tapping (increased negative asynchrony) in the PD group, with fewer delayed responses. The differences between our study and previous studies may be attributed to differences in sensorimotor function of the upper and lower limbs and participant characteristics (9, 41, 42, 53–57).

The CV of the ITI in the Bi-anti condition was significantly larger in the PD group than in the HC group, particularly at an ISI of 1,200 ms, indicating greater rhythm variability. In contrast, the CV in the HC group showed no significant differences across the ISIs. As the BG is involved in time processing in the supra-second range and beat-based temporal processing (1–7), this finding may be consistent with impaired central temporal processing involving BG-related networks.

Patients with PD showed greater negative asynchrony and rhythm variability in the Bi-anti condition than in the other phase conditions, whereas HCs showed no such differences. Numata et al. (29) reported that bilateral antiphase movements in young healthy adults (approximately 20–49 years of age) showed significantly smaller rhythm variability than did unilateral and bilateral in-phase movements. Given that participants in our study were older, the difficulties in synchronization—especially in the Bi-anti condition at shorter ISIs (500–900 ms)—may reflect age-related motor decline. Consistent with prior findings (20–23, 58), the HC and PD groups showed decreased accuracy and stability in the Bi-anti condition, suggesting that our approach may serve as a useful indicator for assessing lower-limb rhythm generation.

An important consideration when interpreting the findings regarding the Bi-anti condition is that this condition differed from the Uni and Bi-in conditions not only in the interlimb phase relationship but also in the source of temporal information. Tapping of the dominant foot was directly synchronized with each auditory tone, whereas the non-dominant foot generated intermediate off-beat timing without a direct external cue. Thus, the greater abnormalities observed in the PD group during the Bi-anti condition may reflect a combination of impaired antiphase interlimb coordination and increased reliance on internally generated timing. As internally generated rhythmic timing is impaired in PD, this task structure may have preferentially increased the temporal demands on the PD group. The present design cannot dissociate the contributions of antiphase coordination and internally generated off-beat timing. Future studies directly comparing single- and dual-cue antiphase conditions will be necessary to separate these components.

Previous studies have linked rhythm maintenance in synchronization-continuation paradigms with the severity of gait disturbances, including FOG (18). In this study, exploratory within-PD-group analyses suggested associations between bilateral foot-tapping performance and clinical severity. In the Bi-in condition, the mean STE and CV of the ITI were associated with clinical scores at several supra-second ISIs. In the Bi-anti condition, the regression coefficient was also associated with disease severity; however, the direction of this association differed across ISIs. At 1,800 ms, greater disease severity was associated with more negative regression coefficients, consistent with greater hastening, whereas at 2,400 ms, the UPDRS scores were positively associated with the regression coefficient. Thus, the relationship between disease severity and the temporal evolution of STE appears to be ISI-dependent, rather than reflecting a uniform increase in hastening. These exploratory findings are consistent with previous observations of impaired bilateral coordination in PD (19–23, 58) but require confirmation in a larger sample.

From a clinical perspective, the present findings may be relevant to gait rehabilitation using external rhythmic cues. The Bi-anti condition required the non-dominant foot to generate an intermediate off-beat response without a direct auditory cue, thereby increasing the demand for internally generated timing. External rhythmic cueing provides an explicit temporal reference and may reduce this demand. Thus, the present findings provide a rationale for future studies examining whether lower-limb tapping abnormalities are related to responsiveness to rhythmic auditory cueing. Nevertheless, in this study, we did not directly assess gait performance or the effects of auditory cueing, and the clinical applicability of these findings remains to be established.

Impaired interlimb coordination during locomotor activities in PD has been discussed in relation to abnormalities in rhythmic lower-limb control (59, 60). Rhythmic locomotor control involves interactions among supraspinal motor regions and brainstem–spinal locomotor networks, including central pattern generators (CPGs) (61, 62). However, the present foot-tapping task consisted of voluntary, auditory-guided movements and did not directly measure locomotor CPG activity. Accordingly, the abnormalities observed in this study are more appropriately interpreted as behavioral alterations in sensorimotor synchronization and temporal control, potentially involving cortico-BG and other supraspinal networks. Although bilateral antiphase foot-tapping exhibits distinct rhythmic characteristics in healthy individuals (29), the extent to which CPG-related mechanisms contribute to the present foot-tapping performance remains unknown and requires direct neurophysiological investigation.

This study has some limitations. First, as we employed a synchronized foot-tapping task, whether early tapping in PD coincides with hastening during self-paced tapping remains unclear; further research employing the synchronization-continuation paradigm is required (18). Second, the neural substrates underlying the abnormalities observed in the Bi-anti condition cannot be directly identified from the present behavioral task. In particular, the present findings do not directly demonstrate the involvement of brainstem–spinal CPGs. The observed abnormalities should therefore be interpreted primarily as alterations in voluntary sensorimotor synchronization and temporal control, rather than as direct evidence of CPG dysfunction. A more comprehensive understanding of the underlying neural mechanisms will require neurophysiological studies using techniques such as functional magnetic resonance imaging. Third, the influence of sensory underestimation in PD (63) on rhythm generation warrants further research. Fourth, all patients with PD were evaluated in the ON-medication state, generally approximately 2–3 h after taking their usual antiparkinsonian medication following a meal; however, the exact interval between the most recent medication dose and testing was not strictly standardized across participants. As dopaminergic medication may modulate temporal processing and motor performance (8, 50–52), differences in medication timing may have contributed to interindividual variability. Therefore, the present findings reflect performance under treated conditions and cannot be directly generalized to the OFF-medication state. Fifth, gait performance, including the accuracy and stability of gait rhythm, was not evaluated. Therefore, future studies are warranted to investigate the relationship between foot-tapping performance and gait performance in patients with PD. Finally, the participants in this study were patients with PD with Hoehn–Yahr stage ≤ III, who were able to walk independently; therefore, the findings cannot be generalized to patients with more advanced disease. In addition, the correlation analyses were based on data from only 14 patients because 1 patient had missing data. A sensitivity analysis based on the bivariate correlation model indicated that, with *n* = 14, a two-sided α of 0.05, and a statistical power of 0.80, the minimum detectable correlation was approximately |r| = 0.66. Thus, moderate associations may have remained undetected, increasing the possibility of type-II error. Furthermore, as multiple exploratory correlations were examined without adjustment for multiple comparisons, the reported associations are susceptible to type-I error. Sensitivity analyses excluding the single participant with Hoehn–Yahr stage I preserved the direction of the reported associations, although several correlations no longer reached the nominal significance threshold. Therefore, the findings from the correlation analyses should be regarded as hypothesis-generating and require confirmation in a larger independent cohort with a broader range of disease severity.

In conclusion, this study demonstrated altered temporal accuracy and stability of rhythmic lower-limb movements in patients with PD, particularly during bilateral antiphase foot-tapping. Exploratory analyses also suggested associations between abnormalities in bilateral foot-tapping performance and clinical severity. These findings indicate that bilateral foot-tapping may provide useful behavioral information regarding lower-limb temporal control in PD. However, as gait performance was not directly assessed and the clinical correlations were exploratory, further studies are required to determine how these abnormalities relate to FOG and gait dysfunction.

## Data Availability

The raw data supporting the conclusions of this article will be made available by the authors, without undue reservation.
